# Innovative cardiovascular primary prevention population-based strategies: a 2-year hybrid type 1 implementation randomised control trial (RCT) which evaluates behavioural change conducted by community champions compared with brief advice only from the SPICES project (scaling-up packages of interventions for cardiovascular disease prevention in selected sites in Europe and sub-Saharan Africa)

**DOI:** 10.1186/s12889-021-11443-y

**Published:** 2021-07-19

**Authors:** Delphine Le Goff, Marie Barais, Gabriel Perraud, Jeremy Derriennic, Paul Aujoulat, Morgane Guillou-Landreat, Jean Yves Le Reste

**Affiliations:** 1 EA 7479 SPURBO, Department of general practice, University of Western Brittany, Brest, France; 2 EA 7479 SPURBO, Department of addictology, University of Western Brittany, Brest, France

**Keywords:** Primary prevention, Cardiovascular system, Community participation, Community health-care workers, Motivational interviewing

## Abstract

**Background:**

Cardiovascular diseases (CVD) caused 17.9 million deaths worldwide in 2016, being the world’s leading cause of death. Prevention of CVD in high-income countries is expensive and fails to reach the population at risk. In low-income countries, it is under-developed. The SPICES project implements a community-based program to improve CVD prevention in 3 European countries and 2 Sub-Saharan countries, based on using community champions to effect behavioural changes. In France, the project operates in “Pays Centre Ouest Bretagne” (COB) which is the Central West Brittany area, and a vulnerable, rural setting. The aim of this study is to assess this innovative prevention strategy versus brief advice.

**Methods:**

A two-step RCT hybrid type 1 implementation study will first of all screen a population using the Non-Laboratory INTERHEART Score (NL-IHRS) and will involve health-care students at public events in the COB area until 1000 participants have been recruited. Second, a RCT will be carried out. The research team will contact each participant with an intermediate NL-IHRS in order to include them. Participants will be over 18 years of age and work or live in the COB area. Participants will be equally randomised in two groups. The intervention group will receive brief advice plus behavioural change guidance carried out by community champions. The control group will receive brief advice only. The main objective for the RCT is to assess a difference of at least 15% in the NL-IHRS between the two groups after 24 months. The primary outcome will be analysed with intention to treat. Secondary outcomes for the RCT will be assessed using validated questionnaires: the WHOQOL-BREF, the DASH Q questionnaire, the IPAQ-short; smoking level will be assessed according to the NL-IHRS scoring system; a modified self-declared alcohol consumption questionnaire has been developed and gauges will be used to assess BMI. The implementation strategy will use mixed methods: qualitative research methods and quantitative epidemiological studies.

**Discussion:**

A difference in the mean NL-IHRS of 15% will provide an argument in favour of reorganising prevention policies. A substantial change would favour relocating primary prevention from healthcare professionals to lay people and the community.

**Trial registration:**

Clinical Trials NCT03886064 - the study was recorded on ClinicalTrials.gov, the 22nd of March 2019.

## Background

Cardiovascular diseases caused 17.9 million deaths worldwide in 2016, representing 31% of deaths and being the world’s leading cause of death according to the World Health Organization (WHO) [1]. Cardiovascular diseases encompass a range of conditions. Among them, myocardial infarction and strokes are the most frequent conditions. More than 75% of cardiovascular deaths occur in low- and middle-income countries. Cardiovascular disease contributes to premature death, especially in low- and middle-income countries, and to morbidity and disability resulting in an economic burden on individuals, communities and countries [2].

Cardiovascular diseases are the result of a combination of modifiable and non-modifiable cardiovascular risk factors. Non-modifiable risk factors include family medical history, age, gender, ethnic background. Modifiable risk factors include medical conditions, such as hypertension, diabetes, excess weight, hypercholesterolemia, chronic renal failure and modifiable behaviours, such as physical inactivity, smoking and unhealthy diet. There is now a growing recognition of the role of stress and depression in contributing to the development of cardiovascular disease [3]. Non-pharmacological interventions to change unhealthy behaviours are effective in improving modifiable risk conditions and, consequently, lowering the occurrence of cardiovascular diseases [4].

Cardiovascular risk assessment cannot be achieved by taking each risk factor individually nor by simply adding them together. Cardiovascular risk assessment has been validated through the development of risk scores, such as the Framingham score or the SCORE which is currently recommended in primary prevention by the European Society of Cardiology [5]. These scores need blood samples from individuals to integrate cholesterol levels which could be difficult for low-income countries to achieve. The Non Laboratory INTERHEART (NL-IHRS) which measures adiposity distribution instead of cholesterol levels has been developed to address this pitfall [3].

As drug therapy has transformed the prognosis of patients with established cardiovascular disease, high-income countries have focused their primary prevention strategies on the prescription of drugs for patients at cardiovascular risk and on regular biological check-ups and medical follow-up. These primary prevention strategies are not as effective as expected and require financial and human resources that middle- and low-income countries cannot afford [6] [7].

Sub-Saharan countries have already faced public health challenges, such as the HIV/AIDS epidemic. They had to find a way to overcome their limited resources to stop the epidemic. One innovative alternative solution was the recruitment of community champions, individuals who were established and acknowledged in their communities and who endorsed peer leadership. They were enrolled to encourage their neighbours to do HIV testing, to accept drugs when necessary and to accept regular medical check-ups. Based on this experience, the role of community champions and the professional role of healthcare workers spread, tackling communicable diseases in the first instance and then non-communicable diseases [8]. As a result, the WHO has developed the Innovative Care for Chronic Conditions framework. This framework helps authorities to adapt local healthcare policies, to embed the care system in the community, to focus care on the patient and his or her family, to support patients in their communities and to focus on preventive care.

The Scaling-up Packages of Interventions for Cardiovascular disease prevention in selected sites in Europe and Sub-Saharan Africa SPICES Study implementation phase (SPICES study) is led by a consortium of six universities from five countries around Europe and Sub-Saharan countries: Belgium, France, United Kingdom, South Africa and Uganda. The aim of this consortium is to develop non-pharmacological cardiovascular prevention interventions in each setting, to evaluate their progress, their barriers and facilitators, at each stage, by using implementation protocols [9]. The consortium focuses on vulnerable populations in each country. Periodic meetings are being held over the five-year duration of the project to share local lessons drawn from the experience of implementation.

In France, the project is held in the “Pays Centre Ouest Bretagne” (COB), a rural setting of 100,000 inhabitants located in the centre of the Brittany region. The COB area was chosen because of the vulnerability of its population. The area has several phone and web access issues due to its isolation. The population of this area was defined as vulnerable because of an excess of overall mortality and cardiovascular mortality [10], a lower average income than in France as a whole, a lower medical density of 8.9 general practitioners per 10,000 inhabitants (compared with 9.9 for Brittany as a whole) and 1.2 consultants per 10,000 inhabitants (compared with 7.1 for Brittany as a whole) [11], a higher suicide rate and a higher level of alcohol consumption [12]. To address these issues, locally elected politicians introduced an unprecedented initiative to the French administrative system. In 2012, they signed the first French healthcare local contracts linking local stakeholders to healthcare administration [13]. Moreover, the country is linked by a network of numerous, dynamic cultural and sports clubs and groups.

Seven French national preventive plans existed in 2018 which partly addressed cardiovascular prevention. There was no connection between the plans. A policy review and qualitative interviews with healthcare professionals from the COB were conducted before defining this protocol [14]. In many instances, a gap persisted between national media campaigns and local initiatives. COB stakeholders and healthcare professionals felt left out of national policies.

The COB area could benefit from the Sub-Saharan experience with their community champions. Local stakeholders were ready to involve new individuals in prevention. It appeared that a space had opened up for local behavioural change programs run by COB community champions. It became possible to identify people at risk of cardiovascular disease and to recruit them for the behavioural change program by involving students who were carrying out their national service in healthcare. The national health service was created in France in 2018 [15]. This service, of 6 weeks duration, is mandatory for medical students, pharmacists, dentists, midwives, physiotherapists and nurses. Students have to be involved in prevention interventions, preferably focused on a vulnerable population. These criteria apply to the SPICES project in France.

Mobile-health (m-health) is experiencing a massive diffusion worldwide and seems a promising means of supporting innovative prevention programs [16]. A discussion was begun on how to integrate m-health tools in the COB which would address the lack of healthcare professionals and the difficulty of data study collection, taking into account the web access issues in the area.

The SPICES project could bridge the gap between national prevention policies and pragmatic grounded prevention. This gap was perfectly identified by the local stakeholders and there seemed to be a strong motivation to bridge this gap. Moreover, the results of the SPICES project could lead to more pragmatic national and international policies.

This article presents the protocol of the French Spices study. It follows the Standard Protocol items: recommendations for Interventional Trials (SPIRIT) 2013 guidelines.

## Methods/design

### Aim of the study

The primary objective is to evaluate the efficacy of a behavioural change program plus brief advice, conducted by community champions, for people at intermediate cardiovascular risk, compared with brief advice only, measuring a 15% decrease of the NL-IHRS score after a 24-month intervention [3].

The secondary objectives are identification of intervention barriers and facilitators at the different phases of the study: during screening and recruitment, during the intervention, according to screeners, community champions, participants, and the research team.

### Eligibility criteria

Two groups of participants will be approached.

The first group will belong to the general population and will be screened with the NL-IHRS. These people will be over 18 years of age and work or live in the COB.

The second group will be composed of people at moderate cardiovascular risk, according to the NL-IHRS (range 9 to 15) and will be recruited for the behavioural change intervention. The research team will only focus on people at intermediate cardiovascular risk because interfering with the habits of healthy people could worsen their existing behaviour. Another consideration is that the provision of exclusive non-pharmaceutical intervention for people at high cardiovascular risk is at odds with current French cardiovascular recommendations.

Non-inclusion criteria for the people undergoing the screening are: aged under 18, current pregnancy, living and working outside COB, personal history of cardiovascular disease.

Non-inclusion criteria for participants undergoing the RCT are: low cardiovascular risk according to the NL-IHRS (score strictly under 9), high cardiovascular risk according to the NL-IHRS (score strictly over 15).

### Interventions

**Fig. 1:**
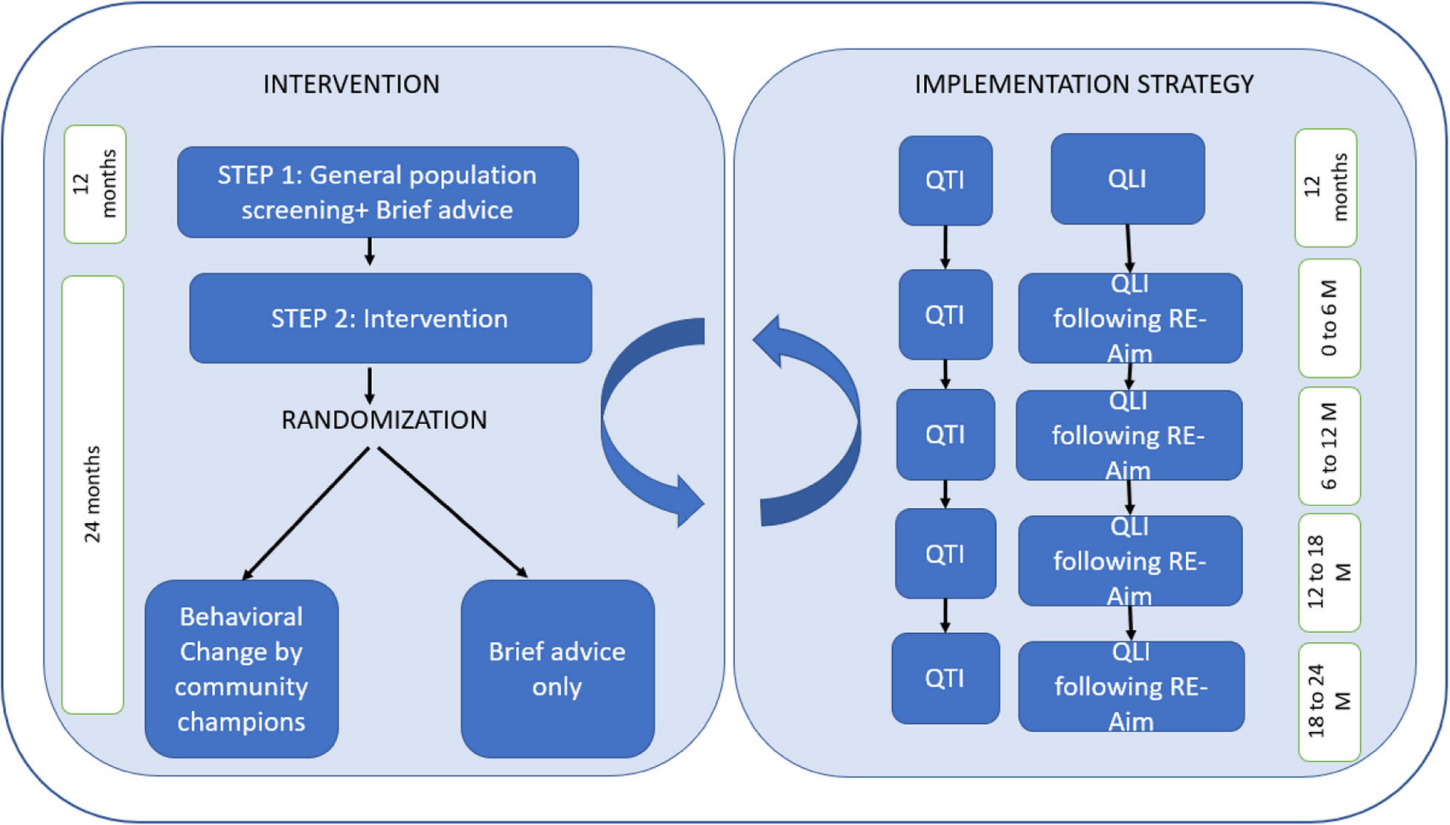
Study Design. *QTI Quantitative Implementation, QLI Qualitative Implementation*

A hybrid type 1 implementation randomised control trial will be conducted with a combination of quantitative and qualitative approaches. The intervention by community champions will be assessed by a randomised control trial (RCT) (Fig. 1).

### STAGE 1: general population screening and brief advice

Extensive screening of the population will be conducted, involving students undertaking national health service. They will use the NL-IHRS developed for use on a tablet, using the REDcap© software. REDCap© allows data collection in the field followed by secured internet transfer of the data. Standardised, appropriate, brief advice will be constructed following the items of the NL-IHRS score, based on the French healthy lifestyle recommendations edited by the Haute Autorité de Santé and Santé Publique France. The brief advice regarding smoking habits and stress and depression will be constructed following the expertise of the team’s addiction expert (MG). Each piece of advice generated will be standard but will only appear on the tablet when it fits the participant’s answer to the particular item in the score.

The screening study setting will be local festive events and medico-social organisations. Access to these events and organisations will be possible following a standard procedure. First, the Community Health Project Manager will identify a referent in each town of the COB area and obtain from that referent a list of the events and organisations likely to welcome screeners. Then the research team will contact the manager of each event or organisation to request an invitation and agree on dates, times, number of screeners needed and the number of persons expected at the event. All invitations will be collected and placed on a global schedule allowing the research team to allocate a junior researcher and a number of screeners to each event. Other voluntary medical organisations will be integrated into the screening phase on request to the research team. The number of events and organisations will be modified until the research team is able to recruit 1000 participants for the next stage. The list of events and organisations will be kept by the research team in accordance with the SPIRIT guidelines.

Every screener will be trained one full day of training. The training will include testing the tablets, the NL-IHRS, communication training and information about ethics research. Junior researchers will receive one full day of training on the same items plus screeners’ guidance.

The content installed on the REDcap© software will include inclusion and exclusion criteria, the NL-IHRS score and the appropriate brief advice.

In the field, screeners will canvass participants, assess the NL-IHRS and deliver the appropriate brief advice. When they have screened someone at intermediate cardiovascular risk, they will offer the participant inclusion in the second stage. Participants willing to be included will give their name, phone number, e-mail address and postal address in a separate sheet, collected by the junior researchers. Participants at low risk will be given the brief advice and positive reinforcement. Participants at high risk will be given the brief advice and be strongly recommended to get an appointment with their usual physician. Screeners will be supported in the field by the junior researchers who will refer to the research team if there is a problem.

Implementation in this stage will include quantitative evaluation, described in the statistical analysis chapter. Qualitative implementation will focus on barriers and facilitators related to screening, as perceived by the screeners, conducted using either focus-groups or individual interviews. A purposive sample of screeners will be created. The sampling criteria will include age, number of participants screened and location of the screening. Data collection will continue until theoretical saturation of the data is reached. A thematic analysis will be performed. Two researchers, working blind, will code the data then merge their analyses.

### Stage 2: RCT

After the completion of stage 1, the research team will contact each participant at intermediate cardiovascular risk to include those participants in the intervention stage. Participants will be randomised in the intervention group, e.g. behavioural change conducted by community champions or in the control group e.g. brief advice only.

First, the community champions will be trained in communication skills improvement based on motivational interviewing coaching. The first version of the training will be appraised by national experts in behavioural change and motivational interviewing and by members of the research team. Champions and members of the research team will try this version, comment on it and propose areas of improvement. The modified version of the training will be used for all the champions. The champions will be followed up individually and in groups from the beginning to the end of the study. Individual training for new champions is planned in the event of withdrawal. Champions will be assigned to geographical areas close to the residential areas of the participants.

Second, RCT intervention group participants will benefit from repeated brief advice every 6 months and motivational group sessions conducted by community champions. Each group session will last one and a half hours. Groups are expected to consist of 10 to 15 participants. The groups will meet 13 times during the 2 years of the study. During a first intensive phase, the groups will meet every 15 days (Day 0, D15, D30, D45, D60). Then, 2 meetings will be scheduled during the 2-month short maintenance phase (M3, M4); finally, a long maintenance phase with meetings every 3 months will last until the end of the study (M7, M10, M13, M16, M19, M21). Champions will use Motivational Interviewing techniques and adopt a support role in the group. Individuals in the group will work on behavioural micro-objectives and micro-changes. The study setting will be community halls loaned by local stakeholders. The research team will set up health conferences at least once, and up to four times, during the study for the participants, depending of the needs expressed by the community champions.

RCT control group participants will benefit from repeated brief advice every 6 months. Brief advice will be delivered as in the intervention group.

Implementation at this phase comprises quantitative and qualitative evaluation. Qualitative evaluation will comprise a profiling of the community champions, focusing on barriers and facilitators relating to follow-up, as perceived by the champions and the research team, by either focus-groups or individual interviews. A purposive sample of screeners will be created. Sampling criteria will include age, number of participants in group, location of follow-up. Data collection will continue until theoretical saturation of the data is reached. A thematic analysis will be performed. Two researchers, working blind, will code the data then merge their analyses.

#### Outcomes

The primary outcome will be the comparison of the NL-IHRS between the two branches of the study after 24 months.

The secondary outcomes after 24 months will be quality of life, as assessed by the WHOQOL-BREF [17], modification of diet, according to the DASH questionnaire [18], modification of physical activity, following the IPAQ-short [19], BMI reduction, smoking level, modification of self-declared alcohol consumption.

- The WHOQOL-BREF questionnaire was derived from the WHOQOL-100 in 1996 to assess quality of life by using thirty-three questions within the four following domains: physical health, psychological domain, social relationships and environment.
- The DASH questionnaire was created in 2016 to assess, by means of eleven questions, the quality of an individual’s diet, according to the Dietary Approach to Stop Hypertension. This diet was designed along the lines of the Mediterranean diet to improve the cardiovascular risk profile of the consumers [20].
- The IPAQ was created in 2003 to assess physical activity. The short version comprises seven questions in four blocks which describe intense activity, moderate activity, walking in the last 7 days and time spent seated. The score of the IPAQ-short can be converted in Metabolic Equivalents of Task, a standardised measurement of physical activity level.

The tertiary outcomes will be the same as previously stated, assessed at 6 and 12 months. The implementation strategy will be assessed by mixed methods comprising qualitative research methods and quantitative descriptive epidemiological studies [21]. Quantitative implementation data collected at 12 and 24 months will consist of the number of people screened with the NL-IHRS, the number of people entering the behavioural change intervention, the number of people completing the entire intervention, the cost of the intervention. Qualitative studies will be constructed using the Re-Aim model [22], exploring invention barriers and facilitators and perspectives from screeners, community champions, stakeholders and members of the research team.

#### Sample size

The primary outcome will be analysed with intention-to-treat. An inter-group comparative analysis will be performed at the end of the intervention on the primary outcome. A difference in the mean NL-IHRS of 15% is expected between the intervention and the control groups. Secondary outcomes at 24 months will consist of differences in scores for quality of life (WHOQUOL-BREF), DASH questionnaire, IPAQ-short, reported smoking status, reported alcohol consumption. Tertiary outcomes, at 6- and 12-months, will consist of differences in the NL-IHRS, WHOQUOL-BREF, DASH questionnaire results, as well as the IPAQ-short, the reported smoking status and the reported alcohol consumption results.

The implementation outcomes will be the number of participants entering the groups, the number of people following the entire intervention (attendance at 75% or more of the group meetings), and the cost of the intervention.

Randomisation will be applied automatically by Redcap©.

### Data collection and management

Data collection methods will use an electronic data recording system using Redcap© on mobile apps. Data storage will be on Redcap©.

Data management and data quality will be assessed by the data management unit of the CHRU Brest.

### Statistical methods

For stage 1, a descriptive epidemiological analysis will be carried out on the screened population. The total number of screenings, the distribution of the population within low-, intermediate- and high-cardiovascular risk scores, and the frequency of the modifiable risk factors listed in the score will be described.

For stage 2, the calculation of the number of subjects required is based on the comparison of the mean NL-IHRS between the two branches of the study: for a power of 80%, an alpha risk = 0.05 and an effect size (mean difference / standard deviation) of 0.20, the number of subjects required is 394 per branch. The figure of 985 subjects, including about 20% potentially lost to follow-up, should be included in the total. With the number of subjects being 394 patients per branch and the hypothesis of an effect size of 0.20 for each secondary endpoint, the test power is approximately 68% for each secondary endpoint using the Holm-Bonferroni correction to allow for multiple comparisons.

The analysis of the RCT will be conducted on an “intention to treat” basis.

No intermediate analysis will be conducted because this study leads to no biomedical risk.

A hierarchical analysis will be conducted in two steps. First, a comparison of the mean NL-IHRS between the two branches at alpha risk = 0.05 then, in the event of a statistically significant result, a comparison of the 6 secondary endpoints between the two branches, using the Holm-Bonferroni correction to consider the multiple comparisons made at this stage. In accordance with the principles of hierarchical analysis, the tests provided for in the second step will only be carried out in a demonstrative manner if the test performed in the first step is statistically significant (*p* < 0.05).

The quantitative outcomes will be compared between the two branches using a Student test. The qualitative endpoints will be compared between the two groups using a Chi-square test. Secondary, multivariate analyses will be performed using a linear model to adjust for potential confounding factors.

Missing data will be identified via the electronic CRF, and investigators will be called back to complete the data. The remaining incomplete files will be declared as lost to follow-up.

### Methods: monitoring

Data monitoring will be overseen by a monitoring committee independent of the sponsors from the CHRU Brest. This will take place at each 6-monthly stage.

### Ethics and dissemination

Ethics approval was obtained from the national French ethics committee. Informed consent will be written for each participant, community champion, screener and researcher.

The final dataset will be hosted via Redcap at The University of Antwerp. The access to the final dataset will be granted by requiring it to the investigators. There is no contractual agreement that limits access to the dataset. A publication policy was designed by the SPICES consortium comprising a project logo, international and national websites, newspapers articles, newsletters, conferences, videos addressed to study participants, stakeholders, and general population.

## Discussion

The aim of this study is to assess an innovative prevention strategy for cardiovascular diseases. If the NL-IHRS is improved, this can provide an argument in favour of reorganising healthcare policies in high-income countries. A substantial change would be to relocate primary prevention from healthcare professionals to lay people and to the community.

If the NL-IHRS is not improved, new hypotheses should be tested. Current evaluation of prevention shows that healthcare professional-led individual cardiovascular primary prevention is not effective. If community-led individual cardiovascular primary prevention targeting behavioural change is not effective either, then public health institutions should readdress budgets directed towards other strategies. Other individual strategies that have been tested include food labelling and economic incentives to promote healthy diet [5]. Public policies that could be effective include public space adaptation, such as the development of pedestrian paths or cycle paths to promote regular physical activity [23].

Secondary aims would be to assess the efficacy of Motivational Interviewing by a RCT which would provide evidence at Level II. Evidence of effectiveness of Motivational Interviewing is still scarce [24]. If Motivational Interviewing is assessed and shown to be effective in this study, it should advocate extensive training among both healthcare professionals and lay people. Group dynamics is also evaluated in this study. A successful outcome would contribute to the upgrading of recommendations edited by national and international guidelines which are scarce and currently supported by a poor level of evidence.

The efficacy of brief advice by professionals is already established for smoking cessation [25]. This study will demonstrate the feasibility of delivering cardiovascular-related brief advice on a large scale.

Additionally, the effectiveness of community champions on cardiovascular prevention will be tested in a high-income country. Initially, the use of community champions was promoted and proved effective in the prevention and treatment of HIV in Sub Saharan countries [26]. The WHO recommends relying on patients’ families and communities when dealing with the burden of chronic diseases [27]. The success of the champions-led intervention would advocate a commitment to involving lay people in cardiovascular prevention. This commitment is both cost-effective and could be disseminated rapidly, compared with the recruitment and training of healthcare professionals. Healthcare costs are a current issue across the board, from low-income countries to high-income countries.

The objectives of this study are ambitious, especially as regards the study duration. A current challenge in changing behaviours and lifestyle habits is the possibility of relapse. Relapse often occurs 6 months to 1 year after the initiation of the change. A two-year duration will produce stronger proof of efficacy. Moreover, many studies did not assess the improvement of cardiovascular risk, as this study will do, only levels of readiness to change, activation of behavioural change or health awareness.

The implementation provides documented understanding about the effectiveness of the intervention and about what works, or does not work, in a peculiar type of setting. The research team chose internationally acknowledged models to structure implementation, such as Re-Aim and CFIR [28, 29]. Implementation tools will be regularly discussed and chosen by consensus within the international team. This study will provide valuable data for future research or prevention implementations, in practical barriers and facilitators related to large population screening and community-led primary prevention. Current French evaluation mainly focuses on barriers to information diffusion on a national scale. This study will demonstrate that the collective involvement of local elected representatives, researchers and local volunteers enables primary prevention programs on a regional scale.

Each country of the SPICES consortium will adapt non-pharmacological cardiovascular prevention interventions to its setting, will evaluate and monitor its progress, using community reinforcement, community champions and community health workers. Data from the French setting will be compared with data from the other SPICES settings, providing an international overview of community-led primary prevention strategies.

## Data Availability

Not applicable.

## Abbreviations

RCT: Randomized controlled trial
SPICES: Scaling-up packages of interventions for cardiovascular disease prevention in selected sites in Europe and Sub-saharan Africa
CVD: Cardiovascular disease
COB: Pays centre ouest bretagne
NL-IHRS: Non-laboratory INTERHEART score
BMI: Body mass index
WHO: World health organization
SPIRIT: Standard protocol items: recommendations for interventional trials
re-aim: Reach, effectiveness, adoption, implementation, and maintenance
CFIR: Consolidated framework for implementation research
